# The Effect of Clear Aligners on Root Length in Endodontically Treated Teeth: A Systematic Review of Split-Mouth Studies

**DOI:** 10.3390/healthcare13182311

**Published:** 2025-09-16

**Authors:** Nefeli Katanaki, Ioanna Pouliezou, Nikolaos P. Kerezoudis, Iosif Sifakakis

**Affiliations:** 1 Division of Orthodontics, University of Geneva, 1205 Geneva, Switzerland; nkatanaki@gmail.com; 2 Medical Research Methodology Unit, School of Medicine, Aristotle University of Thessaloniki, 54124 Thessaloniki, Greece; 3 Department of Orthodontics, School of Dentistry, National and Kapodistrian University of Athens, 2 Thivon Street, 11527 Athens, Greece; isifak@dent.uoa.gr; 4 Department of Endodontics, School of Dentistry, National and Kapodistrian University of Athens, 2 Thivon Street, 11527 Athens, Greece; nkerez@dent.uoa.gr

**Keywords:** orthodontic treatment, clear aligners, endodontic treatment, root resorption, adverse effects, systematic review

## Abstract

**Background/Objectives**: Clear aligners are increasingly prescribed for orthodontic treatment, primarily in adult patients; however, concerns have been raised that this treatment approach may negatively impact root length, especially in endodontically treated teeth. The present investigation aims to systematically synthesize available research addressing the potential effects of clear aligner orthodontic treatment on root length changes in endodontically treated teeth. **Methods**: Four electronic databases were searched until May 2025, and lists of references from relevant publications were screened to identify studies (randomized clinical trials, controlled clinical trials, and observational studies) written in the English language with no date restriction. Clinical studies comparing clear aligner orthodontic treatment in endodontically treated teeth versus vital pulp teeth in humans, using cone beam computed tomography or panoramic radiographs to evaluate root resorption, were assessed. Following study selection and data extraction, the risk-of-bias assessment was evaluated with the Newcastle–Ottawa tool for the observational studies. **Results**: A total of 173 studies were retrieved, and ultimately 2 observational cohort studies were included in the systematic review, encompassing 135 patients (69.6% female; with an average age of 22.5 years). The present review found an association between endodontic status and root resorption, with vital pulp teeth (VPT) exhibiting a greater degree of resorption compared to root canal treated teeth (RCT). Clear aligner (CA) orthodontic treatment resulted in less root resorption than fixed orthodontic appliances (FAs). **Conclusions**: Limited evidence indicates that clear aligner orthodontic treatment leads to a lower occurrence of root resorption and fewer cases of severe root resorption in endodontically treated teeth. Based on findings from studies comparing CAs to FAs, there is overall significantly greater resistance to root resorption in RCT than VPT, irrespective of the orthodontic treatment modality (CAs or FAs).

## 1. Introduction

### 1.1. Rationale

Orthodontically induced root resorption (OIRR), also known as external apical root resorption (EARR) in the literature, involves the loss of root dentin and cementum [1,2]. OIRR specifically refers to the resorption occurring as a consequence of orthodontic treatment, where forces are applied to move teeth. While EARR can have various causes, OIRR is a recognized side effect of orthodontic treatment. In rare, severe cases, this resorption can compromise the crown-to-root ratio and may lead to tooth mobility or loss [1]. It is characterized radiographically by the shortening of the root apex due to loss of hard tissue [3]. Histological studies have concluded that OIRR may affect more than 90% of teeth. As a typical unintended consequence of orthodontic treatment, the occurrence of OIRR is difficult to predict and prevent [1].

OIRR is categorized into three degrees of severity based on the histological findings: firstly, cemental or surface resorption, typically followed by remodeling; secondly, dentinal resorption, often observed in conjunction with reparative process; and thirdly, circumferential apical root resorption, which culminates in root shortening [4].

OIRR represents a potential sequela of orthodontic tooth movement and may affect any tooth; nevertheless, the maxillary and mandibular incisors are consistently identified as the most predisposed to this condition [5]. OIRR’s pathophysiology stems from sterile inflammation within the periodontal tissues, which subsequently progresses to hyaline degeneration, in response to mechanical stress [6]. OIIRR is an inflammatory, dose-dependent response to force systems for tooth movement, modulated by mechanical (force magnitude, duration) and individual risk factors (genetics and anatomy). It can be aggravated by factors like prior trauma or anatomical susceptibility, and is essentially unavoidable, varying only in degree and severity [7]. Under conditions of excessive stress, apoptosis of cementoblasts occurs, thereby exposing the root surface and rendering it vulnerable to odontoclastic activity, particularly within the apical region [8].

The resorptive mechanism is mediated by the coordinated activity of osteoblasts, osteoclasts, odontoblasts, and odontoclasts, and is further regulated by cytokines and hormones [9,10]. Cytokines are locally increased in response to a stimulus, and T-cells are stimulated while expressing RANKL; thus, pre-odontoclasts are activated and differentiated. Odontoblasts and fibroblasts cooperate with bioactive neuropeptides. The expression of RANKL is stimulated by cytokines, including tumor necrosis factor alpha (TNF-α), interleukin-β (IL-β), and IL-6, as well as prostaglandin E2 and hormones generated by the devitalizing periodontal ligament and fibroblasts, which contribute their chemotactic, vasoactive, and cellular effects [11].

In most orthodontic cases, OIRR is clinically insignificant. Rarely, severe OIRR may result in an undesirable crown-to-root ratio and negatively impact the functionality and aesthetics of the orthodontic treatment’s outcome [12,13,14,15,16]. Orthodontic tooth movement, provokes a multifactorial interaction that influences the susceptibility to OIRR, like genetics, age, severity of malocclusion, nutritional habits, type of appliance used, characteristics of force applied (magnitude, direction, duration), treatment approach (extraction vs. non-extraction) [17,18], duration of active treatment [17,18,19,20], bone density and morphology, root shape, and existing/previous trauma [19].

According to recent studies that evaluated panoramic radiographs, root canal treated teeth exhibited lower OIRR compared to vital pulp teeth (VPT), highlighting the need for further investigation [21,22,23,24,25,26]. Some studies also found a correlation between the quality of endodontic treatment and the difference in OIRR in RCT and VPT [27]. Contrary to these findings, other studies have found no significant difference between RCT and VPT in terms of OIRR [28]. Another study even recommended that during orthodontic treatment, it might be effective to perform endodontic procedures to treat or prevent OIRR [29].

Cone beam computed tomography (CBCT) overcomes the limitations of geometric distortion, poor visualization of root structures, and tooth overlap, minimizing incorrect landmark identification and measurement inaccuracy while providing high-resolution, distortion-free imaging to detect minimal values of OIRR [30,31].

The use of fixed appliances (FAs) in orthodontic treatment has been associated with OIRR [32,33]. On the contrary, the impact of clear aligners (CAs) on OIRR remains under investigation. Although CAs are considered advantageous on a theoretical level in reducing the OIRR due to lighter and more controlled force application, current evidence presents inconsistent findings [34].

Several studies confirm the possibility of OIRR with CAs, including cases of severe resorption [35] and a reduction in the crown-to-root ratio [36]. The distance of tooth movement and force transmission characteristics of CAs can affect the occurrence of OIRR. CAs show exponential force decay over time, while auxiliaries like attachments and power ridges have higher forces or moments corresponding to the direction of movement (for premolar derotation, molar distalization, and incisor torque, respectively) [37]. Several systematic reviews underscore that although technological advancements on material and geometric design characteristics have improved CA predictability and tooth movement efficacy, more sophisticated force-tracking technologies are needed to better predict tooth movement outcomes and minimize side-effects, such as OIIRR [38,39]. Further studies comparing CA and FA treatments have yielded inconsistent results. While some suggest that CAs may lead to less OIRR due to gentler forces [14,40,41], others report comparable levels of OIRR in both treatment methods [42,43]. The present inconsistency highlights the need for additional research to elucidate the effects of CA treatment on root resorption.

### 1.2. Objectives

This systematic review aims to critically appraise the available scientific evidence on the impact of CA orthodontic treatment on the root length of RCT.

## 2. Materials and Methods

### 2.1. Protocol and Registration

This systematic review adhered to the methodological framework outlined in the Cochrane Handbook for Systematic Reviews of Interventions, version 6.5. [44]. The reporting process complied with the PRISMA statement (Preferred Reporting Items for Systematic Reviews and Meta-Analysis) [45]. The protocol for this systematic review was prospectively registered in PROSPERO (International Prospective Register of Systematic Reviews) under the registration number CRD420251040487 (https://www.crd.york.ac.uk/prospero/display_record.php?ID=CRD420251040487, accessed on 25 April 2025). The evaluation process strictly followed the registered protocol, with no deviations from the prespecified design.

### 2.2. Eligibility Criteria

The inclusion and exclusion criteria were defined in alignment with the PECOS framework, Participants, Exposure, Comparisons, Outcomes, and Study Design (Table 1).

#### 2.2.1. Types of Participants

The review included healthy human patients presenting with any type of malocclusion and without a prior history of systemic or periodontal disease. No restrictions were applied with respect to age, gender, or ethnicity. The review specifically included patients with RCT undergoing orthodontic treatment with CAs.

#### 2.2.2. Types of Exposures

The exposure in the present review was defined as the orthodontic treatment with CAs in RCT.

#### 2.2.3. Comparisons

Untreated contralateral side (split-mouth). VPT with no orthodontic tooth movement, VPT under orthodontic treatment with FAs, and RCT under orthodontic treatment with FAs.

#### 2.2.4. Types of Outcome Measures

Root resorption of RCT after orthodontic treatment (OIRR/EARR).

#### 2.2.5. Study Design

Randomized Controlled Trials (RCTs), controlled clinical trials (CCTs), and observational studies, including cohort and case–control designs, were deemed eligible for inclusion. Studies employing split-mouth, two-arm, and multi-arm methodologies were also considered. In multi-arm studies, only the experimental groups that fulfilled the eligibility criteria of this systematic review were included. No restrictions were applied regarding the duration of follow-up.

#### 2.2.6. Exclusion Criteria

Individuals presenting with compromised oral health and/or insufficient dental hygiene, as well as patients with systemic conditions that may influence orthodontic tooth movement, were excluded. Additionally, studies on treatments not involving clear aligners were excluded too, as well as studies that did not report at least one of the primary outcomes of interest, such as root resorption, effectiveness of tooth movement, prognosis, or treatment complications. This review excluded in vitro, histological, animal studies, case reports, case series, non-clinical studies, literature reviews, systematic reviews, abstracts only, technique description articles, and opinion pieces.

### 2.3. Information Sources

A comprehensive search of four electronic databases was conducted, without restrictions on publication year or type, from database inception to May 2025: PubMed, Scopus, Embase, and ProQuest. The reference lists of eligible records and existing systematic reviews were reviewed to identify any additional studies.

### 2.4. Search Strategy

Two examiners (N.K., I.P.) conducted the electronic database search by the use of appropriate medical subject headings (MeSH) and free text words. This review included only studies published in English, without date restrictions. Further information regarding the complete electronic search strategy is available in Supplementary Table S1. Gray literature sources, including theses, dissertations, and unpublished studies, were retrieved by ProQuest. Reference lists of the included studies were manually reviewed to detect any further relevant studies not retrieved through the primary database search.

### 2.5. Study Selection Process

All databases and registers were cross-checked to eliminate duplicate records. The selection process was subsequently undertaken in two phases. Initially, two reviewers (N.K., I.P.) independently screened the titles and abstracts of studies retrieved from all electronic databases. In the second phase, full-text articles were independently assessed for eligibility by the same two reviewers (N.K., I.P.) to determine eligibility. Disagreements regarding full text screening and inclusion at this stage were resolved through consensus, with the involvement of the third reviewer (I.S.).

### 2.6. Data Collection and Data Items

Two reviewers (N.K., I.P.) independently extracted data with a standardized, pilot-tested form. Disagreements were resolved through discussion; if consensus was not reached, a third reviewer (I.S.) was consulted. The data extraction form included the following items: general study information: first author, publication year, clinical setting, country, and journal; study characteristics: study design and follow-up duration; participant characteristics: sample size, sex, age, number of RCT and VPT, and malocclusion status; intervention details: type of orthodontic appliance (CA) and treatment protocols; comparator: contralateral untreated side or FA therapy in VPT or RCT; outcomes: primary (root resorption of RCT) and secondary outcomes (effectiveness of tooth movement, prognosis, and treatment complications), as well as methods of outcome assessment (e.g., CBCT and panoramic radiograph); and risk of bias for observational studies, which was independently assessed using the Newcastle–Ottawa Scale tool [46].

### 2.7. Risk of Bias Assessment in Included Studies

The risk of bias of the included observational studies was independently assessed by two reviewers (N.K., I.P.) using the Newcastle–Ottawa Scale (NOS) tool [46]. Disagreements between the two authors were resolved through discussion until a consensus was achieved with the contribution of a third author (I.S.). For each included study, the overall risk of bias was determined according to the highest level of bias across the assessed domains. Based on the NOS, studies were subsequently categorized as being of good, fair, or poor methodological quality. In line with the NOS, scores of 7–9 stars indicate good quality, 4–6 stars indicate fair quality, and 0–3 stars indicate poor quality.

### 2.8. Effect Measures and Data Synthesis

The primary outcome of this systematic review was the effect of CAs on root resorption of RCT. From each eligible study, the following data were extracted: authorship and year of publication, study design, sample size, type of intervention, treatment comparison, method of outcome assessment, reported outcomes, and follow-up period. The intra-reviewer and inter-reviewer reliability regarding the study selection, data collection, and risk of bias assessment was assessed using Cohen’s Kappa statistical tests.

A qualitative synthesis of the findings was pre-specified to be conducted if considerable heterogeneity in clinical or methodological characteristics was identified among the included studies.

## 3. Results

### 3.1. Study Selection

**Figure 1:**
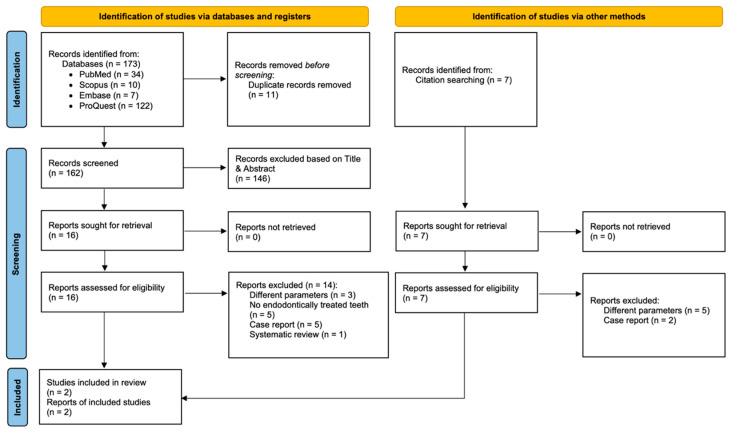
PRISMA 2020 flow diagram.

A total of 173 records were identified across the four databases. Following the removal of 11 duplicate references, 162 articles were thoroughly screened. Of these, 146 were excluded after title and abstract assessment as unrelated to the research topic. After the initial screening, 16 potentially related studies remained for further examination. Subsequently, 14 completed studies, after reviewing the full texts, were excluded in accordance with the eligibility criteria (Supplementary Table S2). Citation searching yielded seven additional records, none of which, however, were eligible. After the full screening process, two articles were deemed eligible and included in the qualitative analysis. The PRISMA2020 flow diagram is illustrated in Figure 1.

### 3.2. Study Characteristics

Study characteristics of the included studies are reported in Table 2, with further extracted data available in Supplementary Table S3. Both included studies were observational cohort studies with a split-mouth design. One study was retrospectively performed using panoramic radiographs, involving 66 patients treated with FAs in group 1 and CAs in group 2, and RCT was compared with their contralateral VPT [47]. The second study evaluated OIRR in RCT and VPT using a split-mouth design and a three-dimensional CBCT image technique [48].

### 3.3. Risk of Bias Within Studies

Risk of bias assessment for the studies included is summarized in Table 3. Both included studies were observational and, therefore, assessed using the Newcastle–Ottawa scale (NOS), to assess the risk of bias, evaluate the selection and comparability of cohorts, and assess outcomes and exposure ascertainment. In line with NOS scoring, values of 0–5 indicated a high risk of bias, 6–7 indicated a moderate risk, and those scoring 8–9 points indicated a low risk. Both studies [47,48] exhibited good quality, each receiving a total score of nine stars. Although both studies achieved high scores in most evaluation criteria, the most significantly impacted domain was the assessment of outcome, as there is no available information regarding the blinding of outcome assessors. The assessment of intra-reviewer and inter-reviewer reliability using Cohen’s Kappa value revealed almost perfect agreement (κ = 0.91) during the processes of study selection, data collection, and risk of bias assessment.

### 3.4. Synthesis of Results

From the two studies, both compared OIRR between RCT and contralateral VPT using a split-mouth design. Liu et al. [48] utilized CBCT and a 3D image registration protocol to assess OIRR volumetrically, whereas Kurnaz and Buyukcavus [47] implemented panoramic radiographs to evaluate root length changes.

A quantitative synthesis (meta-analysis or any form of data synthesis) was not feasible due to both the limited number of eligible studies and the high degree of clinical and methodological heterogeneity among them. Specifically, the included studies differed considerably in terms of design, study populations, and methods of outcome assessment, which would have made any attempt to synthesize the results—whether through forest plots or estimation of a pooled effect size—inappropriate and potentially misleading rather than informative. In accordance with the Cochrane Handbook’s recommendations, we therefore opted for a qualitative (narrative) synthesis to provide an appropriate summary of the available evidence. Consequently, as no meta-analysis could be performed, the application of the GRADE (Grading of Recommendations Assessment, Development, and Evaluation) framework to assess the certainty of evidence was not appropriate.

### 3.5. Results of Individual Studies

Both of the included studies focused on evaluating OIRR by comparing RCT with contralateral VPT in patients undergoing orthodontic treatment with either CAs or FAs.

Liu et al. [48] employed a CBCT-based, three-dimensional image registration technique to assess both volumetric and surface root changes before and after orthodontic treatment. According to reported outcomes, RCT exhibited significantly less OIRR compared to VPT (0.44 ± 0.57 mm vs. 0.67 ± 0.73 mm, *p* = 0.0033, paired *t*-test). Positive correlations were noted between OIRR and treatment duration in both RCT (r = 0.5506, *p* < 0.0001) and VPT (r = 0.4371, *p* = 0.0002), whereas root movement distance demonstrated lower yet statistically significant (SS) positive correlations between and OIRR in both RCT (r = 0.2955, *p* = 0.0140) and VPT (r = 0.2790, *p* = 0.0206). Regarding appliance type, FAs resulted in significantly greater root resorption than CAs in RCT and VPT (*p* < 0.05). More specifically, the FA group demonstrated 0.53 ± 0.57 mm of OIRR in RCT and 0.84 ± 0.64 mm in VPT (*p* < 0.05), while the CA group showed only 0.02 ± 0.36 mm and 0.14 ± 0.62 mm in RCT and VPT, respectively, with no SSD between them (*p* = 0.6016 > 0.05). Intergroup comparisons revealed significantly higher resorption in both RCT and VPT treated with FAs compared to those treated with CAs (*p* < 0.05).

No SSD in OIRR were found between males and females within either RCT (*p* = 0.3434) or VPT groups (*p* = 0.7977), nor in the overall gender-based comparison (*p* = 0.7358). Age was not significantly associated with OIRR within either group (RCT: r = −0.1377, *p* = 0.2592; VPT: r = −0.0504, *p* = 0.6809). However, age demonstrated a strong positive correlation with the difference in OIRR (dOIRR) between RCT and VPT groups (r = 0.8362, *p* = 0.0254).

Subgroup analyses revealed significantly reduced OIRR in RCT compared to VPT for specific tooth types, including premolars (0.19 ± 0.60 mm vs. 0.70 ± 0.52 mm; *p* < 0.05), and for specific tooth position, mainly regarding upper teeth (0.34 ± 0.50 mm vs. 0.68 ± 0.69 mm; *p* < 0.05). Treatment duration was strongly and positively correlated with OIRR in both RCT (r = 0.5506, *p* < 0.0001) and VPT (r = 0.4371, *p* = 0.0002), although no significant correlation was found with the dOIRR between RCT and VPT (r = −0.0641, *p* = 0.6). Additionally, OIRR showed a statistically significant correlation with root movement distance in both RCT (r = 0.2955, *p* = 0.0140) and VPT (r = 0.2790, *p* = 0.0206).

The study by Kurnaz & Buyukcavus [47] utilized panoramic radiographs to assess root length changes in mandibular molars treated with either CAs or FAs. Their findings indicated that RCT exhibited significantly less OIRR compared to VPT within both appliance groups. In the FA group, RCT showed 0.46 ± 0.32 mm of resorption, while VPT exhibited 1.21 ± 0.86 mm (*p* < 0.001). Correspondingly, in the CA group, RCT showed 0.28 ± 0.41 mm of OIRR versus 0.78 ± 0.59 mm of OIRR in VPT (*p* < 0.001). Moreover, CA treatment resulted in significantly lower levels of OIRR compared to FAs in both RCT (*p* < 0.01) and VPT (*p* < 0.01). No SSD were reported with respect to gender or tooth type (*p* > 0.05). RCT are less susceptible to root resorption during orthodontic tooth movement compared to VPT, and no study reported resorption of potential clinical importance in either group.

## 4. Discussion

### 4.1. Summary of Evidence

This systematic review included two retrospective split-mouth studies that evaluated adverse outcomes on root length associated with CA orthodontic treatment. Combined data from two studies and a total of 135 patients demonstrated that RCT exhibited greater resistance to OIRR than VPT, regardless of the orthodontic modality applied (CAs or FAs). This observation may represent a unique biological response in RCT, potentially attributed to the absence of vital pulp, which may alter the transmission of orthodontic forces and mitigate the risk of root resorption.

The included studies, by Kurnaz & Buyukcavus [47] and Liu et al. [48], differed in terms of patient age and imaging methodology. Kurnaz and Buyukcavus [47] investigated younger patients, with a mean age of 18.33 ± 1.96 years for the CA group and 17.45 ± 2.67 for the FA group, utilizing panoramic radiographs for radiographic assessment. In contrast, Liu et al. examined adult participants with a mean age of 27.19 ± 6.08 years and employed three-dimensional CBCT-based image superimposition to assess volumetric changes in root structure. Both studies implemented a split-mouth design, enabling direct contralateral comparisons of RCT and VPT within the same individual, thus strengthening internal validity. However, the discrepancy in diagnostic imaging may have affected the accuracy and precision of OIRR measurement, as panoramic radiographs are associated with greater distortion and lower sensitivity compared to CBCT.

More specifically, Kurnaz & Buyukcavus [47] reported significantly lower OIRR in RCT than in VPT based on panoramic radiographs. Moreover, patients treated with CAs exhibited less resorption than those treated with FAs. Their findings confirmed that both endodontic status and appliance type significantly influenced the amount of resorption observed. No SS associations were found regarding gender or tooth type.

Liu et al. [48], utilizing CBCT, ratified these findings by identifying significantly reduced resorption in RCT compared to VPT. Furthermore, their analysis revealed that increased treatment duration and greater root movement distances were associated with more severe resorption, whereas age, gender, and tooth type did not exhibit a significant association with the occurrence of resorption [48]. While some researchers suggest that advancing age may elevate the risk of root resorption due to reduced vascularity of the periodontal ligament and increased bone density [49], others have reported no significant correlation between chronological age and the extent of root resorption [3,50].

It is crucial to acknowledge the methodological contrast between the two studies. The three-dimensional assessment by CBCT in the study by Liu et al. [48] provides higher accuracy in detecting root surface changes compared to two-dimensional panoramic imaging used by Kurnaz and Buyukcavus [47]. Thus, the difference in imaging modalities for root resorption detection may affect the perception of the resorption, while advanced imaging may offer more reliable insights.

Root resorption is most commonly observed in patients undergoing treatment with orthodontic appliances as a consequence of tooth movement [4,51]. Specifically, the EARR, which involves the loss of apical root tissue, is the form of resorption usually associated with orthodontic tooth movement [17,52]. The incidence of root resorption, notably, appears increased in the maxillary and mandibular incisors as a result of tooth movement [30,31,53]. Although most of the resorption lacks clinical significance, during orthodontic treatment, it is critical for the orthodontists to identify root resorption, considering that these alterations are, in most cases, irreversible [31,54], with severe root resorption posing a risk to teeth’s longevity [31,55].

Liu et al. [48] showed that longer treatment duration and increased root movement distance were positively correlated with the extent of OIRR in both tooth types (RCT and VPT), suggesting that greater tooth movement may be associated with an increased severity of root resorption. Previous studies suggest that the severity and the incidence of OIRR were lower in cases treated with CAs than in those treated with FAs [40,41,56,57,58]. Similar findings are documented in previous systematic reviews [59,60,61,62,63]. Although none of these studies specifically assessed OIRR before and after treatment for CAs, and considering the differences in indications, treatment duration, and biomechanical principles between CAs and FAs, a direct interpretation of OIRR caused by CAs remains unclear [63]. This effect can be observed, possibly due to intermittent, light forces or shorter treatment durations in simple orthodontic cases [59]. Conversely, some studies have reported no significant difference in root resorption rates between treatment with CAs and FAs [42,64]. One should always bear in mind that aligners are not so effective in treating specific orthodontic anomalies; i.e., in some cases, the accuracy of predicted root movement may differ from the actual movement achieved. In these cases, FAs are the appliance of choice, which may precisely control root movement [65].

Additionally, to assess the extent of OIRR, previous research has predominantly relied on periapical or panoramic radiographs, which may introduce distortion and lead to overestimation or underestimation of resorption [66,67,68]. While Kurnaz and Buyukcavus [47] demonstrated reduced prevalence and severity of OIRR in CA-treated patients, and a reduced resorption in RCT, utilizing panoramic radiographs, Liu et al. [48] employed CBCT-based, three-dimensional image registration for volumetric and surface assessments of root changes, confirming the same hypothesis: significantly lower OIRR in RCT compared to VPT and significantly less resorption in patients treated with CAs than those treated with FAs.

In the current literature, the difference in the amount of root resorption between RCT and VPT remains controversial [25,26,69]. While some studies indicate that RCT exhibits reduced root resorption compared to VPT, others report no SSD between the two [25,26,69,70]. A recent systematic review and meta-analysis support that RCT and VPT do not demonstrate significantly different root resorption patterns during orthodontic tooth movement with fixed appliances [71].

The observed effects should also be interpreted in the context of underlying biological mechanisms. Root resorption is a multifactorial process involving cementoblast and odontoclast activity, inflammatory mediators, and mechanical stress [9,10]. Experimental studies have demonstrated that excessive or continuous orthodontic force can trigger localized hyalinization and clastic cell recruitment, leading to root structure loss [72]. Clear aligners, despite delivering forces in a more intermittent and distributed manner, may still trigger stress-mediated cellular responses on teeth with compromised structural or periapical integrity, thus potentially influencing susceptibility to resorption. [73,74].

Some studies suggest that teeth with a history of trauma have a risk for pulp necrosis following orthodontic treatment [75,76]. This risk was confirmed by a recent systematic review published in 2023, based on moderate certainty of evidence, which further recommended that endodontic treatment should be initiated when pulp necrosis is clinically and radiographically confirmed [77]. Other papers, which focused on alterations in pulpal blood flow or the detection of inflammatory markers in teeth extracted for orthodontic purposes, concluded that the inflammatory response in the pulpal tissues is more pronounced in the initial stages of tooth movement. However, this response is often transient [78,79,80,81].

### 4.2. Limitations

Despite its strengths, this review has several methodological limitations, including variability in study designs, heterogeneity in outcome measures, and the small number of eligible studies, which restricts the generalizability of our findings. Specifically, the included studies were retrospective in design and presented inherent limitations, despite their split-mouth design, which aimed to minimize inter-subject variability. Additionally, the limited number of included studies represents an essential limitation of this systematic review. The inclusion of only two studies restricts the generalizability of the findings and reduces the strength of any conclusions drawn. A small sample of studies also limits the ability to explore potential sources of heterogeneity and to conduct subgroup analyses or meta-analyses. This highlights the necessity for further high-quality research to provide more robust evidence.

Several potential confounders should be acknowledged. Factors such as tooth type, age, type of tooth movement, baseline root morphology, history of trauma, systemic conditions, and differences in imaging modalities between the included studies may have influenced the extent of apical root resorption observed; thus, they represent possible sources of bias.

The use of different radiographic modalities, such as panoramic imaging in the study by Kurnaz & Buyukcavus [47] and CBCT in the study by Liu et al. [48], may have potentially affected the accuracy and comparability of root resorption measurements. This is identified as a key methodological limitation and is noted also in the risk of bias assessment, since “assessment of outcome” was the most significantly impacted domain, emphasizing its influence on overall study quality. It should be noted that, although CBCT provides higher diagnostic precision, the increased radiation exposure and cost limitations can affect its clinical applicability.

The anatomical regions to be assessed differed, with Kurnaz & Buyukcavus [47] focusing solely on mandibular molars, while Liu et al. [48] evaluated all tooth types, which may have resulted in variability due to differences in root morphology and varying patterns of movement.

The retrospective design in both studies can possibly introduce the possibility of selection bias and important confounding variables like age and detailed root anatomy. Although SSD were reported in both included studies, confidence intervals were not reported in Kurnaz & Buyukcavus [47], reducing the reproducibility of the findings.

Although Kurnaz & Buyukcavus [47] reported no SS association with tooth type, their sample included mandibular molars, which may have been influenced by root resorption differently from other tooth types. As far as the risk of bias assessment of the included studies is concerned, tools such as ROBINS-I [82] or the Newcastle–Ottawa Scale could be applied and may yield complementary insights.

A meta-analysis was not feasible in the present systematic review due to the methodological heterogeneity of the included studies. This fact highlights the need for further research with more homogeneous study designs, which will allow for a quantitative synthesis of the results. CBCT imaging may enhance the detection and measurement accuracy of root resorption; however, higher costs, radiation exposure, and clinical feasibility considerations may limit its routine use in clinical practice. Moreover, prospective standardized protocols that control for potential confounding variables, either through study design or statistical adjustment, and a longer follow-up period would enhance comparability and allow for meta-analytic synthesis in future systematic reviews.

## 5. Conclusions

Limited evidence indicates that CA orthodontic treatment leads to a lower occurrence of root resorption and fewer cases of severe root resorption in endodontically treated teeth. According to research comparing CAs to FAs, there is overall significantly greater resistance to root resorption in root canal treated teeth (RCT) than vital pulp teeth (VPT), irrespective of the orthodontic treatment modality (CAs or FAs). Consequently, the absence of vital pulp in RCT may induce a distinct biological response to orthodontic forces, potentially reducing the risk of root resorption.

The current body of evidence remains insufficient to warrant changes in clinical protocols. While the available studies provide valuable preliminary insights, their limited number and methodological heterogeneity prevent firm conclusions. Clinicians should therefore be cautious when prescribing clear aligners for endodontically treated teeth until future evidence becomes available. Further high-quality, low-bias clinical trials should be conducted to allow for reliable conclusions regarding the adverse effects of CAs associated with orthodontic treatment on RCT.

## Figures and Tables

**Table 1:** PECO(S) framework.

Component	Definition in This Review
P—Participants	Healthy human patients with malocclusion, without prior systemic or periodontal disease; including patients with RCT undergoing orthodontic treatment. No restrictions regarding age, gender, or ethnicity.
E—Exposure	Orthodontic treatment with CAs in RCT:
C—Comparisons	Untreated contralateral side (split-mouth); VPT with no orthodontic tooth movement; VPT under orthodontic treatment with FAs; RCT under orthodontic treatment with FAs.
O—Outcomes	Primary: Root resorption of RCT after orthodontic treatment (OIRR/EARR). Secondary: Effectiveness of tooth movement, prognosis, treatment complications.
S—Study design	Randomized Controlled Trials (RCTs), controlled clinical trials (CCTs), observational studies (cohort, case–control). Split–mouth, two–arm, and multi–arm methodologies included.

**Table 2:** Characteristics of the included studies in the systematic review.

Authors, Publication Year, Study Setting	Study Design	Treatment Comparison	Participants Sample Size, Sex, Age (Years)	Malocclusion	Outcome Assessment Method	Outcomes	Follow-Up Period
Kurnaz & Buyukcavus, 2024 [47] Turkey	Retrospective observational study, split mouth	FA group vs. CA group EARR in RFT and VPT	Patients (F/M): 66 (39/27) FA: 37 (21 F, 16 M; mean age: 17.45 ± 2.67 years) CA: 29 (18 F, 11 M; mean age: 18.33 ± 1.96 years) Number of RFT and VPT: Split-mouth, two teeth per patient (RFT and VPT), RFT: 66 VPT: 66 CA/RFT: 29 FA/RFT: 37	Skeletal Class I anomalies, moderate crowding, did not involve extractions Treatment duration: SS longer in FA compared to the CA group ABO discrepancy index: similar baseline difficulty between CA and FA groups.	Digital panoramic radiographs	EARR in RFT: Group FA vs. CA: 0.46 ± 0.32 vs. 0.28 ± 0.41, (*p* < 0.01) EARR in VPT: Group FA vs. CA: 1.21 ± 0.86, vs. 0.78 ± 0.59, (*p* < 0.01) EARR in RFT vs. VPT: FA RFT vs. VPT: 0.46 ± 0.32 vs. 1.21 ± 0.86, (*p* < 0.001) CA RFT vs. VPT: 0.28 ± 0.41 vs. 0.78 ± 0.59 (*p* < 0.001) No SSD between FA and CA groups concerning gender tooth type and chronological age.	Until completion of orthodontic treatment [immediately after debonding; FA group mean treatment duration: 1.96 years (SD ± 0.78 years), CA group mean treatment duration: 1.28 years (SD ± 0.51 years)].
Liu et al., 2025 [48] China	Retrospective observational study, split mouth	RFT vs. VPT FA vs. CA	Patients (F/M): 69 (55 F, 14 M; mean age: 27.19 ±6.08 years) Both RFT and VPT consisted of 19 incisors, 15 premolars, 35 molars Number of RFT and VPT: RFT: 69 (19 incisors, 15 premolars, 35 molars) VPT: 69 (19 incisors, 15 premolars, 35 molars) FA/RFT:56 CA/RFT:13	Involved extractions	CBCT	OIRR in RFT: Group FA vs. CA: 0.53 ± 0.57 vs. 0.02 ± 0.36, (*p* < 0.05) OIRR in VPT: Group FA vs. CA: 0.84 ± 0.64 vs. 0.14 ± 0.62 (*p* < 0.05) OIRR in FA: RFT vs. VPT: 0.53 ± 0.57 vs. 0.84 ± 0.64 (*p* < 0.05) OIRR in CA: RFT vs. VPT: 0.02 ± 0.36 vs. 0.14 ± 0.62 (*p* = 0.6016 > 0.05)	Until completion of orthodontic treatment (immediately after debonding; mean: 30.30 ± 10.65 months).

**Abbreviations:** FA: fixed appliance; CA: clear aligner; EARR: external apical root resorption; M: male; F: female; RFT: root filled teeth; VPT: vital pulp teeth; SSD: statistically significant difference; ABO: American Board of Orthodontics; SS: statistically significant; OIRR: orthodontically induced root resorption; CBCT: cone beam computed tomography.

**Table 3:** Risk of bias of included observational (cohort) studies according to the Newcastle–Ottawa Scale (NOS) tool.

Domain	Reference	Kurnaz & Buyukcavus, 2024 [47]	Liu et al., 2025 [48]
**Selection**	Representativeness of the exposed cohort	b ✵	a ✵
	2. Selection of the non-exposed cohort	a ✵	a ✵
	3. Ascertainment of exposure	a ✵	a ✵
	4. Demonstration that outcome of interest was not present at the start of the study	a ✵	a ✵
**Comparability**	Comparability of cohorts based on the design or analysis controlled for confounders	a ✵✵	a ✵✵
**Outcome**	Assessment of outcome	b ✵	b ✵
	2. Was follow-up long enough for outcomes to occur	a ✵	a ✵
	3. Adequacy of follow-up of cohorts	a ✵	a ✵
**Overall**		Good quality	Good quality

Newcastle–Ottawa Scale score: good quality: 7–9 ✵ (stars); fair quality: 4–6 ✵; and poor quality: 0–3 ✵.

## Data Availability

No new data were created or analyzed in this study. Data sharing is not applicable to this article.

## Supplementary Materials

The following supporting information can be downloaded at https://www.mdpi.com/article/10.3390/healthcare13182311/s1, Table S1: Electronic Search Strategy; Table S2: Excluded studies and the reasons beyond exclusion; Table S3: Extracted data of included studies in the systematic review.

## Abbreviations

The following abbreviations are used in this manuscript:

ABO	American Board of Orthodontics
CAs	Clear Aligners
CBCT	Cone Beam Computed Tomography
CCTs	Controlled Clinical Trial
dOIRR	Difference in Orthodontically Induced Root Resorption
EARR	External Apical Root Resorption
F	Females
FAs	Fixed Appliances
M	Males
OIRR	Orthodontically Induced Root Resorption
RCT	Root Canal Treated Teeth
RCTs	Randomized Controlled Trials
SS	Statistically Significant
SSD	Statistically Significant Difference
VPT	Vital Pulp Teeth
