# Supplementary material for: The Effect of Clear Aligners on Root Length in Endodontically Treated Teeth: A Systematic Review of Split-Mouth Studies
Source: Healthcare (Basel). 2025 Sep 16;13(18):2311. doi: 10.3390/healthcare13182311 (PMC12469331; doi:10.3390/healthcare13182311)
Supplement: Supplementary file 1 [file healthcare-13-02311-s001.zip › healthcare-3784234-supplementary.pdf]

Supplementary Materials

Table S1. Electronic Search Strategy.

Database	Search Strategy	Results
**PubMed**	("Root canal treat*" OR endodontic* OR "endodontically treated teeth" OR "non vital teeth") AND ("clear aligner*" OR Invisalign)	34
**Scopus**	TITLE-ABS-KEY(("root canal treat*" OR endodontic* OR "endodontically treated teeth" OR "non vital teeth" OR nonvital) AND ("clear aligner*" OR invisalign))	10
**Embase**	("endodontic*":ab,ti,kw,de OR "non-vital tooth":ab,ti,kw,de OR "root canal treat*":ab,ti,kw,de) AND ("clear aligner*":ab,ti,kw,de OR invisalign:ab,ti,kw,de)	7
**ProQuest**	("endodontically treated teeth" OR "root canal treated teeth" OR endodontic* OR "non-vital teeth") AND ("clear aligner*" OR Invisalign)	122

*: wildcard operator in database search syntax

Table S2. Excluded studies and the reasons beyond exclusion

Databases	Study	Reason for exclusion
PubMed		
1.	Almalki SA, Al Jameel AH, Gowdar IM, Langaliya A, Vaddamanu SK, Di Blasio M, Cervino G, Minervini G. Impact of clear aligner therapy on masticatory musculature and stomatognathic system: a systematic review conducted according to PRISMA guidelines and the Cochrane handbook for systematic reviews of interventions. BMC Oral Health. 2024 Mar 19;24(1):350. doi: 10.1186/s12903-024-04029-8. PMID: 38504207; PMCID: PMC10949793.	Irrelevant topic - Title/Abstract (T/A)
2.	Kocadereli I, Taşman F, Güner SB. Combined endodontic-orthodontic and prosthodontic treatment of fractured teeth. Case report. Aust Dent J. 1998 Feb;43(1):28-31. doi: 10.1111/j.1834-7819.1998.tb00148.x. PMID: 9583222.	Case report - T/A
3.	Sfondrini MF, Scribante A. Materials and Techniques in Dentistry, Oral Surgery and Orthodontics. Materials (Basel). 2024 Jul 2;17(13):3247. doi: 10.3390/ma17133247. PMID: 38998328; PMCID: PMC11243620.	Irrelevant topic - T/A
4.	Al-Mutairi MA, Al-Salamah L, Nouri LA, Al-Marshedy BS, Al-Harbi NH, Al-Harabi EA, Al-Dosere HA, Tashkandi FS, Al-Shabib ZM, Altalhi AM. Microbial Changes in the Periodontal Environment Due to Orthodontic Appliances: A Review. Cureus. 2024 Jul 12;16(7):e64396. doi: 10.7759/cureus.64396. PMID: 39130947; PMCID: PMC11317031.	Irrelevant topic - T/A
5.	Bucur SM, Moga RA, Olteanu CD, Bud ES, Vlasa A. A Retrospective Study Regarding the Efficacy of Nuvola® OP Clear Aligners in Maxillary Arch Expansion in Adult Patients. Diagnostics (Basel). 2025 Mar 16;15(6):738. doi: 10.3390/diagnostics15060738. PMID: 40150081; PMCID: PMC11941744.	Irrelevant topic - T/A
6.	Ahmad Rasul. Root Resorption in Invisalign vs. Fixed Orthodontic Treatment. United States -- California: Loma Linda University; 2020.	Irrelevant topic - T/A
7.	Singh M, Kaur P. Temporary endodontics during active orthodontics. J Indian Dent Assoc. 1990 Dec;61(12):279, 281, 283. PMID: 2130099.	Irrelevant topic - T/A
8.	Mackie IC, Richmond S, Blinkhorn AS. Trauma of treatment failure: a case report. Endod Dent Traumatol. 1990 Dec;6(6):279-81. doi: 10.1111/j.1600-9657.1990.tb00432.x. PMID: 2094603.	Irrelevant topic - T/A
9.	Jiang W, Wang Z, Zhou Y, Shen Y, Yen E, Zou B. Bioceramic micro-fillers reinforce antibiofilm and remineralization properties of clear aligner attachment materials. Front Bioeng Biotechnol. 2024 Jan 9;11:1346959. doi: 10.3389/fbioe.2023.1346959. PMID: 38318418; PMCID: PMC10840140.	Irrelevant topic - T/A
10.	Taylor J. Metcalf. Comparison of external apical root resorption in Class I subjects treated with Invisalign and conventional orthodontics: A CBCT analysis. United States -- Missouri: Saint Louis University; 2016.	Irrelevant topic - T/A

11.	Bandić R, Vodanović K, Vuković Kekez I, Medvedec Mikić I, Galić I, Kalibović Govorko D. Thickness Variations of Thermoformed and 3D-Printed Clear Aligners. Acta Stomatol Croat. 2024 Jun;58(2):145-155. doi: 10.15644/asc58/2/4. PMID: 39036327; PMCID: PMC11256873.	Irrelevant topic - T/A
12.	Li ZY, Lin MZ, Wang Y, Cai XR, Wang XD, Huang XQ. Effect of endodontically treated teeth on prosthetically guided orthodontics with clear aligners: a case series. BMC Oral Health. 2024 Oct 18;24(1):1242. doi: 10.1186/s12903-024-05007-w. PMID: 39425114; PMCID: PMC11490176.	Case series - T/A
13.	Gabriella Noel Gallo. A Three-Dimensional Comparative Analysis of Root Resorption Among Different Ethnic Groups Throughout Orthodontic Treatment. United States -- Texas: The University of Texas School of Dentistry at Houston; 2024.	Irrelevant topic - T/A
14.	Alajmi S, Shaban A, Al-Azemi R. Comparison of Short-Term Oral Impacts Experienced by Patients Treated with Invisalign or Conventional Fixed Orthodontic Appliances. Med Princ Pract. 2020;29(4):382-388. doi: 10.1159/000505459. Epub 2019 Dec 17. PMID: 31842018; PMCID: PMC7445657.	Irrelevant topic - T/A
15.	Grzebieluch W, Grajzer M, Mikulewicz M. Comparative Analysis of Fused Deposition Modeling and Digital Light Processing Techniques for Dimensional Accuracy in Clear Aligner Manufacturing. Med Sci Monit. 2023 Aug 3;29:e940922. doi: 10.12659/MSM.940922. PMID: 37533235; PMCID: PMC10413909.	Irrelevant topic - T/A
16.	Reid C. Risinger. A Three-Dimensional Comparative Analysis of Root Resorption After Clear Aligner Therapy. United States -- Texas: The University of Texas School of Dentistry at Houston; 2023.	Irrelevant topic - T/A
17.	Šimunović L, Čekalović Agović S, Marić AJ, Bačić I, Klarić E, Uribe F, Meštrović S. Color and Chemical Stability of 3D-Printed and Thermoformed Polyurethane-Based Aligners. Polymers (Basel). 2024 Apr 11;16(8):1067. doi: 10.3390/polym16081067. PMID: 38674987; PMCID: PMC11054520.	Irrelevant topic - T/A
18.	Simpson S, Wallace CK, Vernazza CR. Paediatric dentistry provision in the North East of England: workforce confidence and attitudes. Br Dent J. 2022 Mar 18:1–7. doi: 10.1038/s41415-022-4045-9. Epub ahead of print. Erratum in: Br Dent J. 2022 Apr;232(7):474. doi: 10.1038/s41415-022-4148-3. PMID: 35304592; PMCID: PMC8932095.	Irrelevant topic - T/A
19.	D'Arcangelo C, Vadini M, Buonvivere M, De Angelis F. Safe and Ultraconservative Rehabilitation of Worn Teeth Patients: How Sectional Clear Aligners May Enhance the Prosthetic Treatment Plan. Case Rep Dent. 2022 Oct 30;2022:8305893. doi: 10.1155/2022/8305893. PMID: 36349122; PMCID: PMC9637473.	Irrelevant topic - T/A
20.	Liu F, Wang Y, Luopei D, Qu X, Liu L. Comparison of fixed braces and clear braces for malocclusion treatment. BMC Oral Health. 2024 Aug 14;24(1):941. doi: 10.1186/s12903-024-04469-2. PMID: 39143508; PMCID: PMC11323350.	Irrelevant topic - T/A
21.	Raluca Fratila C, Alonso-Ezpeleta LÓ, Poveda-Saenz M, Giovannini G, Lobo-Galindo AB, Flores-Fraile J, Zubizarreta-Macho Á. Accuracy Evaluation of Indirect Bonding Techniques for Clear Aligner Attachments Using 3D-Printed Models: An In Silico and Physical Model-Based Study. Materials (Basel). 2025 Feb 11;18(4):780. doi: 10.3390/ma18040780. PMID: 40004304; PMCID: PMC11857747.	Irrelevant topic - T/A

22.	Kehoe JC. Splinting and replantation after traumatic avulsion. J Am Dent Assoc. 1986 Feb;112(2):224-30. doi: 10.14219/jada.archive.1986.0328. PMID: 3512655.	Irrelevant topic - T/A
23.	Brandon Fowler. A Comparison of Root Resorption Between Invisalign Treatment and Contemporary Orthodontic Treatment. United States -- California: University of Southern California; 2010.	Irrelevant topic - T/A
24.	Ghorbani M, Mousavi SA, Bardideh E, Saeedi P, Shahnaseri S, Shafaee H, Akyalcin S. The Effectiveness of Functional Clear Aligners for Class II Correction in Growing Patients: A Systematic Review and Meta-Analysis. Orthod Craniofac Res. 2025 Feb 26. doi: 10.1111/ocr.12908. Epub ahead of print. PMID: 40008555.	Irrelevant topic - T/A
25.	Shafaee H, Shahnaseri S, Ghorbani M, Bardideh E, Mousavi SA, Akyalcin S. The Use of Clear Aligners for Orthognathic Surgery: A Systematic Review. J Oral Maxillofac Surg. 2025 Mar 20:S0278-2391(25)00177-6. doi: 10.1016/j.joms.2025.03.009. Epub ahead of print. PMID: 40194551.	Irrelevant topic - T/A
26.	Jacobs SG. The treatment of traumatized permanent anterior teeth: case report & literature review. Part I--Management of intruded incisors. Aust Orthod J. 1995 Mar;13(4):213-8. PMID: 8975659.	Irrelevant topic - T/A
27.	Alamri H, Alshammari FR, Bin Rahmah A, Alsaif MI, Almutairi F, Alolaywi H, Altariqi A, Alotaibi S, Almutairi R, Almadhoon H, AlMoharib HS. Evaluating knowledge and awareness of 3D design and printing among dental students in Saudi Arabia: a cross-sectional study. Front Dent Med. 2024 Nov 25;5:1466393. doi: 10.3389/fdmed.2024.1466393. PMID: 39917671; PMCID: PMC11797805.	Irrelevant topic - T/A
28.	Krupińska-Nanys M, Zarzecka J. An Assessment of Oral Hygiene in 7-14-Year-Old Children undergoing Orthodontic Treatment. J Int Oral Health. 2015 Jan;7(1):6-11. PMID: 25709359; PMCID: PMC4336665.	Irrelevant topic - T/A
29.	Boyd RL. Periodontal and restorative considerations with clear aligner treatment to establish a more favorable restorative environment. Compend Contin Educ Dent. 2009 Jun;30(5):280-2, 284, 286-8 passim. PMID: 19514262.	Irrelevant topic - T/A
30.	Zhang Y, Wang K, Li M, Liu C, Tang L, Wan C, Fan C, Liu Y. Effects of different intrusion patterns during anterior teeth retraction using clear aligners in extraction cases: an iterative finite element analysis. Front Bioeng Biotechnol. 2024 Jun 6;12:1388876. doi: 10.3389/fbioe.2024.1388876. PMID: 38903188; PMCID: PMC11186992.	Irrelevant topic - T/A
31.	Oday R, Abid M, Dziedzic A. The accuracy and retention of presurgical infant orthopaedics constructed from different polymer materials: A comparative study. J Taibah Univ Med Sci. 2024 Feb 5;19(2):379-389. doi: 10.1016/j.jtumed.2024.01.005. PMID: 38370166; PMCID: PMC10874750.	Irrelevant topic - T/A
32.	Karaçay S, Gurton U, Olmez H, Koymen G. Multidisciplinary treatment of "twinned" permanent teeth: two case reports. J Dent Child (Chic). 2004 Jan-Apr;71(1):80-6. PMID: 15272664.	Irrelevant topic - T/A
33.	Machorowska-Pieniążek A, Skucha-Nowak M, Mertas A, Tanasiewicz M, Niedzielska I, Morawiec T, Baron S. Effects of Brazilian Propolis on Dental Plaque and Gingiva in Patients with Oral Cleft Malformation Treated with Multibracket and	Irrelevant topic - T/A

	Removable Appliances: A Comparative Study. Evid Based Complement Alternat Med. 2016;2016:2038407. doi: 10.1155/2016/2038407. Epub 2016 Sep 8. PMID: 27672397; PMCID: PMC5031880.	
34.	Liou EJ, Huang CS. Rapid canine retraction through distraction of the periodontal ligament. Am J Orthod Dentofacial Orthop. 1998 Oct;114(4):372-82. doi: 10.1016/s0889-5406(98)70181-7. PMID: 9790320.	Irrelevant topic - T/A
35.	Ashworth-Davies G, Johnson EL, Sharma PK. Transient apical breakdown associated with clear aligner therapy: a case report. Br Dent J. 2025 Jan;238(1):29-32. doi: 10.1038/s41415-024-8115-z. Epub 2025 Jan 10. PMID: 39794574.	Case report - T/A
36.	Mamber EK. Treatment of intruded permanent incisors: a multidisciplinary approach. Endod Dent Traumatol. 1994 Apr;10(2):98-104. doi: 10.1111/j.1600-9657.1994.tb00069.x. PMID: 8062816.	Irrelevant topic - T/A
37.	Jafarzadeh H, Talati A, Basafa M, Noorollahian S. Forced eruption of adjoining maxillary premolars using a removable orthodontic appliance: a case report. J Oral Sci. 2007 Mar;49(1):75-8. doi: 10.2334/josnusd.49.75. PMID: 17429186.	Irrelevant topic - T/A
Scopus		
1.	Ashworth-Davies G, Johnson EL, Sharma PK. Transient apical breakdown associated with clear aligner therapy: a case report. Br Dent J. 2025 Jan;238(1):29-32. doi:10.1038/s41415-024-8115-z.	Case report - T/A<br>DUPLICATE [PubMed #30]
2.	Li ZY, Lin MZ, Wang Y, Cai XR, Wang XD, Huang XQ. Effect of endodontically treated teeth on prosthetically guided orthodontics with clear aligners: a case series. BMC Oral Health. 2024 Oct 18;24(1). doi:10.1186/s12903-024-05007-w.	Irrelevant topic - T/A<br>DUPLICATE [PubMed #10]
3.	Tietmann C, Jepsen S, Kauer R, Jepsen K. Clinical effectiveness of regenerative periodontal surgery and orthodontic tooth movement with clear aligners in stage IV periodontitis: a case series. Quintessence Int. 2024 May 30;55(5):348-57. doi:10.3290/j.qi.b5231521.	Irrelevant topic - T/A
4.	Alamri H, Alshammari FR, Rahmah AB, Alsaif MI, Almutairi F, Alolaywi H, et al. Evaluating knowledge and awareness of 3D design and printing among dental students in Saudi Arabia: a cross-sectional study. Front Dent Med. 2024 Nov 25;5:1466393. doi:10.3389/fdmed.2024.1466393.	Irrelevant topic - T/A<br>DUPLICATE [PubMed #22]
5.	Li XL, Xu L, Zhang HH. Analysis of highly cited papers in Chinese core journals of stomatology from 2016 to 2021. Chin J Oral Maxillofac Surg. 2023 May 20;21(3):272-8. doi:10.19438/j.cjoms.2023.03.012.	Irrelevant topic - T/A
6.	Song Z, Fang S, Ma X, Jin Z, Liu Q. A review of the effect of clear aligner therapy on oral microecology. Chin J Orthod. 2022 Dec;29(4):222-5. doi:10.3760/cmaj.cn115797-20220419-22410.	Irrelevant topic - T/A
7.	Parthiban G, Nagesh S, Karale R, Reddy S. Multidisciplinary approach to the management of a subgingivally fractured anterior tooth using an aligner-based esthetic orthodontic extrusion appliance: a case report. Saudi Endod J. 2020 May-Aug;10(2):157-61. doi:10.4103/sej.sej_3_19.	Case report - T/A

8.	Boyd RL. Periodontal and restorative considerations with clear aligner treatment to establish a more favorable restorative environment. Compend Contin Educ Dent. 2009 Jun;30(5):280-2, 284, 286-8.	Irrelevant topic - T/A<br>DUPLICATE [PubMed #24]
Embase		
1.	Ashworth-Davies G, Johnson EL, Sharma PK. Transient apical breakdown associated with clear aligner therapy: a case report. Br Dent J. 2025 Jan;238(1):29-32. doi: 10.1038/s41415-024-8115-z. Epub 2025 Jan 10. PMID: 39794574.	Case report - T/A
2.	Li, Z. Y., Lin, M. Z., Wang, Y., Cai, X. R., Wang, X. D., & Huang, X. Q. (2024). Effect of endodontically treated teeth on prosthetically guided orthodontics with clear aligners: a case series. *BMC oral health*, *24*(1), 1242. https://doi.org/10.1186/s12903-024-05007-w	Case series - T/A<br>DUPLICATE [PubMed #10]
3.	Boyd RL. Periodontal and restorative considerations with clear aligner treatment to establish a more favorable restorative environment. Compend Contin Educ Dent. 2009 Jun;30(5):280-2, 284, 286-8 passim. PMID: 19514262.	Irrelevant topic - T/A<br>DUPLICATE [PubMed #24]
4.	Tietmann C, Jepsen S, Kauer R, Jepsen K. Clinical effectiveness of regenerative periodontal surgery and orthodontic tooth movement with clear aligners in stage IV periodontitis: a case series. Quintessence Int. 2024 May 30;55(5):348-357. doi: 10.3290/j.qi.b5213521. PMID: 38619257.	Irrelevant topic - T/A<br>DUPLICATE [Scopus #3]
5.	Alanazi AK, Alqahtani AM, Asiri AM, et al.. Frequencies of root canal treatment and deep caries in clear aligner compared to conventional orthodontic treatment: a retrospective cohort study. Eur J Mol Clin Med. 2022, 9:8218-23.	Irrelevant topic - T/A
ProQuest		
1.	Johnny Tran. Effect of Clear Aligner Therapy on Masticatory Muscle Tenderness and Orthodontic Pain. Canada -- Ontario, CA: The University of Western Ontario (Canada); 2018.	Irrelevant topic - T/A
2.	Maryam Baneshi. Effectiveness of Clear Orthodontic Aligners in Correcting Malocclusion- Systematic Review. England: The University of Manchester (United Kingdom); 2023.	Irrelevant topic - T/A
3.	Sabrina Dorfmann. The Quality of Information on Oral Hygiene Instructions for Orthodontic Patients in TikTok Videos. United States -- Maryland: University of Maryland, Baltimore; 2023.	Irrelevant topic - T/A
4.	Fatemah Husain. Influence of Invisalign’s Precision Bite Ramps on Deep Bite Correction and Root Length in Adults. United States -- New York: State University of New York at Buffalo; 2023.	Irrelevant topic - T/A
5.	Jiménez, J.,Carlos Eusebio. (2023). *Revisão narrativa de modelos finitos em alinhadores* (Order No. 31003752). Available from ProQuest Dissertations & Theses Global. (2906114450). Retrieved from https://www.proquest.com/dissertations-theses/revisão-narrativa-de-modelos-finitos-em/docview/2906114450/se-2	Irrelevant topic - T/A

6.	Brandon T. S. Knapp. Effect of Build Angle and Print Layer Thickness on Clinical Accuracy of 3D-Printed Orthodontic Models. United States -- Missouri: University of Missouri - Kansas City; 2024.	Irrelevant topic - T/A
7.	Kory Grahl. Effect of Slot Dimensions on Aligner Retention. United States -- Nevada: University of Nevada, Las Vegas; 2024.	Irrelevant topic - T/A
8.	Analia Tahir. Optimizing Orthodontic Clear Aligner Treatment With Artificial Intelligence Driven Dental Monitoring. United States -- Illinois: University of Illinois at Chicago; 2024.	Irrelevant topic - T/A
9.	Erica Shapiro Frenkel. Occlusal Contact Changes in Patients Treated with Clear Aligners. United States -- Washington: University of Washington; 2022.	Irrelevant topic - T/A
10.	Zesheng Chen. Accuracy of Composite Attachment Position with Four Different Indirect Bonding Techniques. United States -- Illinois: University of Illinois at Chicago; 2019.	Irrelevant topic - T/A
11.	Kramer Sherman. Clinical Utilization of Orthodontic Assistants in the United States and Corresponding Regional Review. United States -- Oklahoma: The University of Oklahoma Health Sciences Center; 2019.	Irrelevant topic - T/A
12.	Julian Schwafert. Business Plan: Dental Monitoring. Portugal: ISCTE - Instituto Universitario de Lisboa (Portugal); 2021.	Irrelevant topic - T/A
13.	Louis Wenger. Thermal Properties of Commonly Used Clear Aligner Systems As-Received and After Clinical Use. United States -- Wisconsin: Marquette University; 2017.	Irrelevant topic - T/A
14.	Pheba Abraham. COVID-19, Food Insecurity, and Access to Oral Healthcare. United States -- Illinois: University of Illinois at Chicago; 2023.	Irrelevant topic - T/A
15.	1.Yosmely Altagracia Then Hiciano. Percepção, Satisfação e Qualidade de Vida nos Pacientes Tratados com Alinhadores: Revisão Narrativa. Portugal: Egas Moniz School of Health and Science (Portugal); 2023.	Irrelevant topic - T/A
16.	Kha Vu Nguyen. Buccal Alveolar Bone Changes in Adult Non-Extraction Clear Aligner Patients: A Retrospective CBCT Study. United States -- Washington: University of Washington; 2024.	Irrelevant topic - T/A
17.	Lowe,Ronald J., I.,II. Teleorthodontics: Perception and Reliability of Virtually Obtained Records for Orthodontic Decision Making. United States -- New York: State University of New York at Buffalo; 2023.	Irrelevant topic - T/A
18.	Madison Healy. Maxillary Central Incisor RR in Hispanic and Non-Hispanic White Patients with RME. United States -- California: Loma Linda University; 2022.	Irrelevant topic - T/A
19.	Sophie Ju Hyung Moon. Impact of COVID-19 Induced Closure of Orthodontic Clinic on Clinical Outcomes. United States -- Illinois: University of Illinois at Chicago; 2023.	Irrelevant topic - T/A

20.	Isaac Lucas Chen. Alterations in the Subgingival Microbiome During Orthodontic Treatment. United States -- California: University of California, San Francisco; 2018.	Irrelevant topic - T/A
21.	Benjamin Rush. Extraction vs. Non-Extraction: Comparing Orthodontic Root Resorption. United States -- California: Loma Linda University; 2022.	Different parameters: ORR assed only with fixed appliances (T)
22.	Nicholas Valeri. Differential scanning calorimetry (DSC) analyses of esthetic nickel-titanium wires as-received and after clinical use. United States -- Wisconsin: Marquette University; 2013.	Irrelevant topic - T/A
23.	Hsuan Rebecca Tsuei. Impact of Covid-19 Induced Closure on Orthodontic Outcomes in the State of Illinois. United States -- Illinois: University of Illinois at Chicago; 2024.	Irrelevant topic - T/A
24.	Andrew Christopher Lee. A Comparison of Orthodontic Intraoral Scan Results Between Staff and Volunteers. United States -- Nevada: University of Nevada, Las Vegas; 2020.	Irrelevant topic - T/A
25.	Anuja Kothari. SEM assessment of the enamel surface after debonding of ceramic brackets. United States -- Florida: Nova Southeastern University; 2015.	Irrelevant topic - T/A
26.	Neal Eden Bastian. Mouthguards During Orthodontic Treatment: Perspectives of Orthodontists and a Survey of Orthodontic Patients Playing School-Sanctioned Basketball and Football. United States -- Washington: University of Washington; 2018.	Irrelevant topic - T/A
27.	Zachary Von Smith. Three-Dimensional Assessment of External Apical Root Resorption with Carriere Motion Appliance in Comparison with Class II Elastics. United States -- Missouri: Saint Louis University; 2020.	Irrelevant topic - T/A
28.	Ieva Raščiūtė. Herbsto Aparato Įtaka Atraminių Dantų Šaknų Struktūrai. Lithuania: Lithuanian University of Health Sciences (Lithuania); 2022.	Irrelevant topic - T/A
29.	Lola Marec. Impacto do tratamento ortodôntico sobre o periodonto. Portugal: Egas Moniz School of Health and Science (Portugal); 2022.	Irrelevant topic - T/A
30.	Austyn C. Grissom. Root Surface Changes in Endodontically Treated Teeth Following Orthodontic Movement. United States -- Texas: The University of Texas School of Dentistry at Houston; 2022.	Different parameters: Assessed RR with CAs and fixed together-did not differentiate in results (T)
31.	Wilson David Jo Siu. System Informatics and Point Cloud Analysis for Dental Alignment Treatments. United States -- New York: State University of New York at Buffalo; 2021.	Irrelevant topic - T/A
32.	Reem Meshal Almeshal. Evaluation of Antibacterial Activity and Shear Bond Strength of Orthodontic Adhesive Containing Chitosan Nanoparticles. United States -- Massachusetts: Tufts University School of Dental Medicine; 2022.	Irrelevant topic - T/A

33.	Theodore Eliades. The Release of Compounds and Identification of the Pathways Involved in the Cytotoxicity and Xeno-Estrogenicity of Polymeric Dental Biomaterials Utilized in Orthodontics and Paediatric Dentistry. England: The University of Manchester (United Kingdom); 2019.	Irrelevant topic - T/A
34.	Irish Abigail M. Tongco. Comparative Analysis of Buccal Versus Lingual En Masse Anterior Retraction Techniques Used to Treat Adult Bimaxillary Protrusion. United States -- Missouri: Saint Louis University; 2017.	Irrelevant topic - T/A
35.	Shaeeb Rasid. Evaluation of Modified Microosteoperforation on the Rate of En Masse Retraction. A Prospective Clinical Study. India: Rajiv Gandhi University of Health Sciences (India); 2018.	Irrelevant topic - T/A
36.	Taneisha S. Livingston. Microbiome Shifts in the Supragingival Biofilm in Orthodontic Patients. United States -- North Carolina: The University of North Carolina at Chapel Hill; 2022.	Irrelevant topic - T/A
37.	Christopher J. Kirk. The influence of camera perspective on diagnostic accuracy using orthodontic records. United States -- North Carolina: The University of North Carolina at Chapel Hill; 2016.	Irrelevant topic - T/A
38.	Mary Lauren Sharp. Short Root Anomaly: Prevalence, Genetics, and Its Effect in Orthodontic Treatment. United States -- Alabama: The University of Alabama at Birmingham; 2023.	Irrelevant topic - T/A
39.	Maria Fernanda Petroche. A Classification of Maxillary Premolar Sockets in Relation to the Osseous Housing for Immediate Implant Placement. United States -- Massachusetts: Boston University; 2021.	Irrelevant topic - T/A
40.	Fred Andrew Arino. Robotics in Orthodontics: Efficacy of Cad/Cam-manufactured Archwires in Predicting and Implementing Clinical Outcomes. United States -- Pennsylvania: Temple University; 2020.	Irrelevant topic - T/A
41.	Anuj Anil Deshpande. Dimensional Accuracy Evaluation of 3D Printed Teeth Model for Orthodontics. United States -- New York: State University of New York at Binghamton; 2020.	Irrelevant topic - T/A
42.	Ana Yoleida Suárez Acevedo de. Implicações do COVID-19 em medicina dentária : Uma abordagem multidisciplinar. Portugal: Egas Moniz School of Health and Science (Portugal); 2023.	Irrelevant topic - T/A
43.	Karan Sangiv Patel. Short Term Orthodontic Retention Study Focusing on the Differences in the Ability of Hawley Retainers and Essix Retainers to Settle a Bite following Debonding of Braces. United States -- Missouri: Saint Louis University; 2022.	Irrelevant topic - T/A
44.	Siddhi Jyotindra Doshi. Tensile bond strength of brackets bonded to bleached enamel treated with antioxidants. United States -- Illinois: University of Illinois at Chicago; 2009.	Irrelevant topic - T/A
45.	Karina Carolina Rochabrun Arrieche. Dimensional Changes in 3D Printed Models From Two Different Technologies Under Different Storage Conditions. United States -- Massachusetts: Boston University; 2025.	Irrelevant topic - T/A

46.	Sally Mahmoud Elshennawy. In-Vitro Characterization of the Effects of a Developing Oral Probiotic Against Dental Caries in Orthodontic Patients (Streptococcus salivarius LAB813, a Probiotic Against Dental Caries). Canada -- Ontario, CA: University of Toronto (Canada); 2023.	Irrelevant topic - T/A
47.	Jenevieve L. Walbrecker. Exploring the Utility of Dental Pathological Conditions in Dental Radiographic Comparisons. United States -- Nevada: University of Nevada, Reno; 2023.	Irrelevant topic - T/A
48.	Rita Rodrigues Silva. Mordida Aberta: Diagnóstico Tratamento e Estabilidade. Portugal: Universidade Fernando Pessoa (Portugal); 2014.	Irrelevant topic - T/A
49.	Diana Filipa Silva Marques. Tratamento Ortodôntico em Pacientes Periodontais Revisão Narrativa. Portugal: Universidade de Lisboa (Portugal); 2021.	Irrelevant topic - T/A
50.	Ju-Han Chang. The effect of water storage on bending properties of esthetic, fiber-reinforced composite orthodontic wires. United States -- Wisconsin: Marquette University; 2012.	Irrelevant topic - T/A
51.	Amr Chalabi. The Advancement in 3D Printing Technology and Its Applications with Bone Grafting and Dental Implants. United States -- Massachusetts: Boston University; 2022.	Irrelevant topic - T/A
52.	Khulood K. Alzahrani. Impact of Preparation Designs and Computer-Aided Design/Computer-Aided Manufacturing Materials on the Fracture Resistance of Dental Posterior Indirect Adhesive Restorations. United States -- Iowa: The University of Iowa; 2024.	Irrelevant topic - T/A
53.	Jeffrey W. Lam. A Comparison between Ceph Analysis on Lateral Cephs, CBCT Scans, and MRI Scans. United States -- California: Loma Linda University; 2013.	Irrelevant topic - T/A
54.	Bradford C. Washington. Mechanical Properties of Coated vs. Non-Coated Nickel-Titanium Wires. United States -- Illinois: University of Illinois at Chicago College of Dentistry; 2013.	Irrelevant topic - T/A
55.	Rodney Harwood Jones. An in vitro assessment of orthodontic bracket debonding characteristics and color change of teeth repeatedly bleached with a desensitizing 20 percent carbamide peroxide gel. United States -- Kentucky: University of Louisville; 2002.	Irrelevant topic - T/A
56.	Ronak J. Bhagat. Oral Microbiome and Metabolome in Fixed Orthodontic Therapy. United States -- Iowa: The University of Iowa; 2024.	Irrelevant topic - T/A
57.	Kyle C. Tuttle. Assessment of the Infrazygomatic Crest and Distal Infrazygomatic Crest for Bone Depth and Miniscrew Insertion. United States -- Nevada: University of Nevada, Las Vegas; 2021.	Irrelevant topic - T/A
58.	Matilde Duarte Guerra. Sustentabilidade em medicina dentária e alinhamento com a agenda 2030 da Organização das Nações Unidas. Portugal: Egas Moniz School of Health and Science (Portugal); 2023.	Irrelevant topic - T/A

59.	Noor Tarazi. Pre-doctoral Orthodontic Online Learning Effects on Clinical Decision Making. United States -- Pennsylvania: Temple University; 2020.	Irrelevant topic - T/A
60.	Margarida Parreira Fernandes Cortes Cavaco. Margarida Parreira Fernandes cortes Cavaco. Portugal: Egas Moniz School of Health and Science (Portugal); 2023.	Irrelevant topic -T/A
61.	Pinto,Joana Fontes de Sá Coutinho Álvares Gonçalves. Resposta Óssea à Força de Tração Sobre Mini-Implantes - Um Estudo Piloto na Tíbia de Coelho. Portugal: Universidade do Porto (Portugal); 2021.	Irrelevant topic - T/A
62.	Nikolaos Karpitsaris. Oral-health Related Quality of Life, Self-esteem and Bullying Among Patients Receiving Orthodontic Treatment; Comparison Between Labial and 2D-Lingual Orthodontic Appliances. Turkey: Marmara Universitesi (Turkey); 2019.	Irrelevant topic - T/A
63.	Rowan Softley. Advanced In Vitro Oral Biofilm Modelling: Combining Culture Techniques With Next Generation Sequencing. England: The University of Liverpool (United Kingdom); 2024.	Irrelevant topic - T/A
64.	Anne Littlewood. Developing a Framework for Priority-Setting in Evidence Synthesis: a Case Study of Cochrane Oral Health. England: The University of Manchester (United Kingdom); 2022.	Irrelevant topic - T/A
65.	1.Mona Alenezi. Microleakage of Temporary Restorations in Endodontically Accessed Teeth; Thermocycling, Brushing, and Coating Effects: An in Vitro Study. United States -- Massachusetts: Boston University; 2018.	Irrelevant topic - T/A
66.	Ayinkeran Gunarajasingam. Investigations of Novel Endodontic Sealers and Modifications of Existing Bioceramic Sealers. United States -- Massachusetts: Boston University; 2024.	Irrelevant topic - T/A
67.	Bárbara Sá de Moraes. O Branqueamento Dentário na Atualidade. Portugal: Universidade do Porto (Portugal); 2021.	Irrelevant topic - T/A
68.	Darianna Masih. Enhancing Cervical Composite Restorations Management. United States -- Maryland: University of Maryland, Baltimore; 2024.	Irrelevant topic - T/A
69.	Blake R. Borello. Orthodontic treatment with and without removable aligner systems by general dentists. United States -- Missouri: University of Missouri - Kansas City; 2010.	Irrelevant topic - T/A
70.	Michael K. DeLuke. Orthodontic Treatment Provided by General Dentists and Pedodontists: A National Survey. United States -- Connecticut: University of Connecticut; 2005.	Irrelevant topic - T/A
71.	Marta Agnieszka Tomaszewska. Bond Strength of Calcium Silicate Based Bioceramic Sealers to Dentin. United States -- Massachusetts: Boston University; 2023.	Irrelevant topic - T/A
72.	de Carvalho, Bruno João Pinto. Medicina Dentária Digital – Presente e Futuro. Portugal: Universidade Fernando Pessoa (Portugal); 2013.	Irrelevant topic - T/A

73.	Ashima Sharma. Educational Effect of Virtual Orthodontic Patient Screening in Undergraduate Dental Curricula. United States -- Pennsylvania: Temple University; 2022.	Irrelevant topic - T/A
74.	Doyoung Choi. Comparison of Two Orthodontic Sealants in Prevention of Enamel Demineralization : An In-Vitro and In-Vivo Study. United States -- West Virginia: West Virginia University; 2013.	Irrelevant topic - T/A
75.	Priyanka Chandy. A Study to Assess the Effectiveness of Structured Teaching Programme on Dental Problems and its Management with a View to Improve Self Esteem Among Adolescents in Selected Schools in Mysore. India: Rajiv Gandhi University of Health Sciences (India); 2012.	Irrelevant topic - T/A
76.	Só Pereira. Reabsorção Radicular Apical Externa Associada Ao Tratamento ortodôntico: Fatores De Suscetibilidade genéticos, biológicos e mecânicos. Portugal: Universidade de Coimbra (Portugal); 2014.	Irrelevant topic - T/A
77.	Johnny Gonzalez. Maxillary Incisors Step: Matter of Preference? United States -- Massachusetts: Boston University; 2018.	Irrelevant topic - T/A
78.	Gabriela Rosa. Benefícios da terapia ortodôntica prévia a realização de facetas cerâmicas estéticas. Portugal: Egas Moniz School of Health and Science (Portugal); 2018.	Irrelevant topic - T/A
79.	Debbie M. Parnes. The impact of office design on orthodontic office production: A qualitative assessment of the opinions of doctors, staff, and patients. United States -- Pennsylvania: Temple University; 2011.	Irrelevant topic - T/A
80.	Regis Antonio Farah Simony. Reabsorção radicular em incisivos inferiores no tratamento ortodôntico a: revisão sistemática. Portugal: Egas Moniz School of Health and Science (Portugal); 2021.	Systematic review (Portugese)
81.	de Sá Coelho, António Luis Leão. Launching a New Brand in Dental Care - Assessing the Opportunities and Threats for the Portuguese Market. Portugal: Universidade NOVA de Lisboa (Portugal); 2021.	Irrelevant topic - T/A
82.	Ramandeep Samra. Heritability of Nasal Characteristics Using Lateral Cephalograms. United States -- Massachusetts: Boston University; 2018.	Irrelevant topic - T/A
83.	Antoine Lemoine. Odontology & Artificial Intelligence. Portugal: Universidade Fernando Pessoa (Portugal); 2019.	Irrelevant topic - T/A
84.	Milena Petkova. The Association Between a Dietary Inflammatory Index and Periodontal Disease in the National Health and Nutrition Examination Survey 2009-2014. United States -- Massachusetts: Boston University; 2020.	Irrelevant topic - T/A
85.	Miguel Murteira Pedrosa. Abordagens terapêuticas para o encerramento de diastemas incisivos. Portugal: Egas Moniz School of Health and Science (Portugal); 2017.	Irrelevant topic - T/A
86.	Chad Foster. Orthodontic confidence of senior dental students: A study of 2 US dental schools. United States -- California: University of Southern California; 2010.	Irrelevant topic - T/A

87.	Костадинов, Константин Стойчев (Kostadinov, Kostadinov Stoychev). Multimodal Imaging Documentation in Dental Medicine. Bulgaria: Medical University of Varna (Bulgaria); 2023.	Irrelevant topic - T/A
88.	François Renaud Simon Marie Giard. Instrumentos de sopro, influências na cavidade oral e abordagem terapêutica. Portugal: Egas Moniz School of Health and Science (Portugal); 2020.	Irrelevant topic - T/A
89.	Abdullatif H. Sheshter. Assessment of the orthodontic referral by undergraduate dental students. United States -- Illinois: University of Illinois at Chicago; 2008.	Irrelevant topic - T/A
90.	Lihsin Wu. Effects of Comprehensive Orthodontic Treatment with Distalization of Maxillary Molars on the Mandibular Dentition. United States -- Massachusetts: Boston University; 2020.	Irrelevant topic - T/A
91.	Chelsea Ko-Adams. Quantification of Mutans Streptococci and Persister Cells in Orthodontic Patients: A Pilot Investigation. Canada -- Ontario, CA: University of Toronto (Canada); 2018.	Irrelevant topic - T/A
92.	Samim Taraji. Novel Machine Learning Algorithms for Prediction and Treatment Decision in Patients with Class III. United States -- Illinois: University of Illinois at Chicago; 2022.	Irrelevant topic - T/A
93.	Khalid Jamal Alawadhi. A Pilot Study: Stress Level of Patients in Periodontal Treatment with and without Hypertension. United States -- Massachusetts: Boston University; 2017.	Irrelevant topic - T/A
94.	Haifa Maktabi. Implications of Underperforming Light Energy Delivery for Posterior Composite: Insights into Delivered Radiant Exposure, Degradation Characteristics, and Biofilm Formation. United States -- Maryland: University of Maryland, Baltimore; 2018.	Irrelevant topic - T/A
95.	MaryEvan Shepperd Thacker. Testing the Predictibility of Virtual Setups: A Retrospective Comparison of Posttreatment Clinical Models and Virtual Setups. United States -- Massachusetts: Boston University; 2017.	Irrelevant topic - T/A
96.	Andrey Gaiduchik. Effect of Centric Interference on Canine Tooth Wear. United States -- California: Loma Linda University; 2018.	Irrelevant topic - T/A
97.	Abdulrahman Ghoneim. How Does Competition Affect the Clinical Decision-making of Dentists in Ontario?. Canada -- Ontario, CA: University of Toronto (Canada); 2018.	Irrelevant topic - T/A
98.	James Edward Goglia. Mouthguard Use in NCAA Basketball. United States -- North Carolina: The University of North Carolina at Chapel Hill; 2020.	Irrelevant topic - T/A
99.	Ghassan Al-Ayoub. The effects of machining on the flexural strength of CAD-CAM materials. United States -- Massachusetts: Boston University; 2016.	Irrelevant topic - T/A
100.	de Azevedo e Silva, Rita Maria. Impressões 3D em Diferentes Equipamentos. Portugal: Universidade do Porto (Portugal); 2019.	Irrelevant topic - T/A

101.	Ashlee B. Charnoplosky. Oral Cancer Screening Techniques Utilized by West Virginia Dental Hygienists. United States -- West Virginia: West Virginia University; 2011.	Irrelevant topic - T/A
102.	Matthew E. Durschlag. Prediction of Root Form Using Crown Data: Mandibular Left First Premolar. United States -- California: Loma Linda University; 2017.	Irrelevant topic - T/A
103.	Osama Alsulaiman. The Relationship of Cephalometric and Airway Characteristics in SRBD and Expansion Patients: An Observational Study. United States -- Massachusetts: Boston University; 2021.	Irrelevant topic - T/A
104.	Sarah El Meaoui. Reabilitação oral na era digital. Portugal: Egas Moniz School of Health and Science (Portugal); 2023.	Irrelevant topic - T/A
105.	Kavitha Joy. A Comparative Evaluation of Apical Root Resorption in Maxillary Anterior Teeth in Patients Treated with Twostep and En Masse Space Closure Procedures-A CBCT Study. India: Rajiv Gandhi University of Health Sciences (India); 2019.	Parameters: RR after fixed appliances only
106.	Neil A. Wilson. Prospective Study to Quantify the Bone-Retaining Properties of Para-Immediate Dental Implants in the Maxilla. England: The University of Manchester (United Kingdom); 2008.	Irrelevant topic - T/A
107.	Daniel Nikitas Richmond. The Medical Necessity of Orthodontic Care: A Qualitative Study. Canada -- Ontario, CA: University of Toronto (Canada); 2023.	Irrelevant topic - T/A
108.	Merdan Jumayev. Comparing the Effects of Piezocision and Low-Level Laser Therapy in Orthodontic Canine Distalization. Turkey: Marmara Universitesi (Turkey); 2019.	Irrelevant topic - T/A
109.	Bonnie Yu. Perceived Professional Roles, Moral Communities, Moral Inclusiveness, and Dentists' Treatment Decisions. Canada -- Ontario, CA: University of Toronto (Canada); 2019.	Irrelevant topic - T/A
110.	LaDeane Fattore-Bruno. Use of digital technology and support software programs in the private dental offices in Nevada. United States -- Illinois: University of Illinois at Chicago, Health Sciences Center; 2009.	Irrelevant topic - T/A
111.	Virginia M. Hardgraves. Older Adults' Expectations, and Knowledge of Oral Health Issues: A Study of Arkansas Seniors. United States -- Arkansas: University of Arkansas; 2019.	Irrelevant topic - T/A
112.	Xiaohan Guo. Analysis of Spatial Functional Data With Phase Variation. United States -- Ohio: The Ohio State University; 2022.	Irrelevant topic - T/A
113.	Andreia Patrícia Castro Afonso. Qualidade de Vida Relacionada com a Saúde Oral: Adaptação Linguística e Cultural do Ohip-14. Portugal: Universidade Fernando Pessoa (Portugal); 2014.	Irrelevant topic - T/A
114.	Dennis J. Tartakow. An Analysis Of Factors That Align With Faculty Vacancies In Orthodontic Education. United States -- Ohio: Union Institute and University; 2010.	Irrelevant topic - T/A

115.	Weihao Wang. Ionization-Based Irrigation Activation System in Root Canal Treatment: EndoLogic. United States -- Massachusetts: Boston University; 2024.	Irrelevant topic - T/A
116.	James Noble. Motivations and future life, career and practice plans of orthodontic residents in Canada and the United States. Canada -- Manitoba, CA: University of Manitoba (Canada); 2008.	Irrelevant topic - T/A
117.	Hessa Sulaiman A. Alrejaye. Introduction of Novel CAD/CAM Fabricated Esthetic Interpenetrating Phase Ceramic Orthodontic Brackets; Fracture and Frictional Resistance Evaluation. United States -- Massachusetts: Boston University; 2020.	Irrelevant topic - T/A

Table S3. Extracted data of included studies in the systematic review

Authors, publication year	Subject group	Type and site of intervention/technical aspects of interventions	Orthodontic protocol	Outcome assessment method
Kurnaz and Buyukcavus, 2024<br><br>Turkey	CA vs FA	Split-mouth design. Panoramic Radiographs obtained from patients undergoing OT. Radiographs divided into 2 groups: CA treatment and FA treatment. Measurement on digital PRs before OT and immediately after debonding.<br><br>*Incl. Criteria:* Class I treated/moderate crowding/no extr., at least 1 mand, molar w/RCT at least a year prior, existence of antagonist, no bruxism, same amount of OTM between FAs and CAs groups. ABO discrepancy index.<br><br>• **CA**: 29 patients (18 females, 11 males)<br>• **FA**: 37 patients (21 females, 16 males)<br><br>Amount of EARR: in mand. Molars, both groups	• **CAs:** (ClearCorrect, ClearCorrect LLC, Rock Round, TX, USA), virtual tx planning (ClearPilot - ClearCorrect's Planning Tool, version 5; ClearCorrect). 15-day intervals of CAs/22h wear time/day, Mean tx duration: 1.28 years<br><br>• **FA:** conventional bracket (MBT, 0.022; Razor SS brackets, IOS), NiTi archwires (0.014-, 0.016-, 0.018-, 0.016x0.022-, 0.017x0.025 and 0.019x0.025) for levelling and SS (0.019X0.025) for working phase. Mean tx duration 1.96 years	Digital panoramic radiographs captured pre- and post-OT were used to measure tooth lengths and root surface measurements in mandibular molars. Crown and root measurements obtained using program Castellini X-ray unit (Castellini X Radius Compact, Imola, Italy).<br><br>Key steps (line connecting CEJ-midpoint of two Root apices). Measurement: in mm, ratio of crown/root pre and post. Final EARR measurement by combination of these values.<br>• Kolmogorov-Smirnov test, parametric tests, Pearson correlation, t-test, ANCOVA, SPSS
Liu et al., 2025<br><br>China	RCT vs VPT	Retrospective clinical study. Split mouth design. CBCT. 2 groups: FA and CA. CBCT before OTx and at debonding using<br><br>*Incl. Criteria:* RCT monitored for at least 1 year, no bruxism<br><br>Root movement was compared bet RFT and VPT groups	• **CA:** Invisalign, Align Technology, Santa Clara, Calif)<br><br>• **FA:** 0.022x0.026 passive self-ligating bracket appliance	Cone-beam computed tomography (CBCT): measured changes of root length before and after treatment.<br><br>• ShapiroWilk test, t-test, Student t-test or Wilcoxon test, Anova

or Kruskal, Pearson, R 4.1.1 statistical analysis
